# Coal Fly Ash Derived Silica Nanomaterial for MMMs—Application in CO_2_/CH_4_ Separation

**DOI:** 10.3390/membranes11020078

**Published:** 2021-01-21

**Authors:** Marius Gheorghe Miricioiu, Violeta-Carolina Niculescu, Constantin Filote, Maria Simona Raboaca, Gheorghe Nechifor

**Affiliations:** 1 National Research and Development Institute for Cryogenic and Isotopic Technologies–ICSI Ramnicu Valcea, 4th Uzinei Street, 240050 Ramnicu Valcea, Romania; marius.miricioiu@icsi.ro (M.G.M.); simona.raboaca@icsi.ro (M.S.R.); 2 Faculty of Applied Chemistry and Materials Science, Politehnica University of Bucharest, 1-7 Polizu Street, 011061 Bucharest, Romania; gheorghe.nechifor@upb.ro; 3 Faculty of Electrical Engineering and Computer Science, Stefan cel Mare University of Suceava, 720229 Suceava, Romania; constantin.filote@gmail.com

**Keywords:** coal fly ash, gas separation, mesoporous silica, MMMs

## Abstract

In order to obtained high selective membrane for industrial applications (such as natural gas purification), mixed matrix membranes (MMMs) were developed based on polysulfone as matrix and MCM-41-type silica material (obtained from coal fly ash) as filler. As a consequence, various quantities of filler were used to determine the membranes efficiency on CO_2_/CH_4_ separation. The coal fly ash derived silica nanomaterial and the membranes were characterized in terms of thermal stability, homogeneity, and pore size distribution. There were observed similar properties of the obtained nanomaterial with a typical MCM-41 (obtained from commercial silicates), such as high surface area and pore size distribution. The permeability tests highlighted that the synthesized membranes can be applicable for CO_2_ removal from CH_4_, due to unnoticeable differences between real and ideal selectivity. Additionally, the membranes showed high resistance to CO_2_ plasticization, due to permeability decrease even at high feed pressure, up to 16 bar.

## 1. Introduction

In the last decades the mixed matrix membranes (MMMs) attracted special attention, being studied with different matrix materials and fillers in various compositions [1,2,3,4]. MMMs combine the properties of easy processability of the polymers with the superior selectivity of the inorganic materials [5]. Obtaining MMMs can surpass the difficult and expensive fabrication of inorganic membranes, by using polymers as the continuous matrix and inorganic particles as filler. Generally, the MMMs attracted researcher’s attention due to their scalability and cost-effectiveness, offering the advantage of combining the polymers properties, thus going beyond the upper bound limit, but without notable economic drawback [6]. In this respect, mesoporous silica materials were studied as potential inorganic phases. Among them, MCM-41 or SBA-15 were intensively used due to their characteristics (high specific surface, ordered structure, narrow channels). MCM-41 is a mesoporous silica developed for the first time by Mobil company in 1992, by using cationic surfactants in basic media and 1,3,5-trimethyl benzene as swelling agent [7,8]. SBA-15, having a hexagonal structure of cylindrical mesopores, interconnected by micropores randomly distributed, can be synthesized by using triblock copolymers, such as Pluronic P123 and either tetraethyl orthosilicate (TEOS) or sodium silicate used as silica source, under acidic conditions [9,10]. The SBA-15 presents a better thermal stability than MCM-41 [11]. Contrary to the MCM-41, containing isolated hexagonally ordered mesopores, SBA-15 has interconnecting irregular micropores in the walls of the ordered mesopores, which appeared by flexible amphiphilic polyethylene chains penetration in the silica matrix. This interconnected micro-mesoporous structure and thicker mesopore walls confer a better thermal and hydrothermal stability for SBA-15 compared to MCM-41.

The selective MMMs could be a reliable solution for gas separation/purification if the fabrication and operating costs are low. Furthermore, the obtaining of valuable materials from wastes constitutes a potential solution in waste management. A cheap waste is the fly ash, found in large quantities due to coal consumption [12,13]. Even if, nowadays, the fly ash is used in large quantities in the construction industry, its potential as source of interesting compounds with special properties has not yet been reached.

Therefore, finding a feasible application based on directly used of fly ash or fly ash derived materials could significantly reduce environmental impact due to its disposal.

For example, the fly ash was used directly or modified with alkaline groups for CO_2_ trapper [14,15]. When CO_2_ contacted with the unmodified fly ash, there were observed two phenomena: carbonation reaction (resulting calcium carbonate) and CO_2_ absorption [15]. From the point of view of uptake CO_2_ yield, a sorption capacity of approximately 180 g per kg of dry ash was reached, at 318 K and 15 bar [15]. Additionally, the impregnation of fly ash with amino groups (ethylenepentamine) increased the CO_2_ adsorption capacity up to 25% amine loading, due to chemisorption phenomenon caused by the active sites and then decreased, as a result of low porosity influencing directly CO_2_ diffusion [14].

A milestone in the application of fly ash in CO_2_ adsorption is the regeneration of the materials, which is typically influenced by temperature, heating rate, and flow rates [14,16]. Thus, it was demonstrated that a maximum temperature of 473 K (with an increment of 10 K/min) is sufficient for a complete regeneration of the ash in about 30 min [16], while, in other studies the temperature was below 423 K and the time shorter [14]. This difference could be explained by the applied gas flow rates. When the fly ash was functionalized with amino groups, the regeneration was achieved at a temperature below 393 K, in order to avoid the amine disruption [14].

Various studies proposed silica synthesis from coal fly ash, due to its high amount of SiO_2_ and Al_2_O_3_. The silica can be further used, directly or as a filler in a polymer matrix, for CO_2_ adsorption or various pollutants’ retention from wastewater [17,18,19]. It was observed that, not only the amount of Si in fly ash and its recovery are important, but also the chemical and mineralogical composition of the derived material [12]. Thus, it is necessary to further investigate the influence of various types of fly ash, with determined Si/Al ratios in the resulted supernatant, on the properties of the obtained silica materials [12].

Therefore, this study aimed to evaluate the obtaining MMMs based on polysulfone as a polymer matrix and MCM-41 silica filler derived from fly ash, with direct application in CO_2_ removal from natural gas, in order to solve two environmental issues (fly ash management and CO_2_ reduction).

## 2. Materials and Methods

### 2.1. Obtaining Mesoporous MCM-41

In order to obtain mesoporous silica, the ash must be converted into silicate solution. The synthesis of the mesoporous silica material followed a previous developed procedure [20]. Briefly, 1 g fly ash was dried, crushed, screened, sifted, then it was mixed with 1.2 g NaOH, reagent grade, ≥98% (Sigma Aldrich, Darmstadt, Germany) and treated at 973 K for 30 min. The formed products were then dissolved in ultrapure water and placed in a Teflon autoclave at 378 K for 24 h. The supernatant resulting after filtration was further used as silica source.

In order to obtain the mesoporous MCM-41, a validated synthesis was applied [21,22]. Briefly, 60 mL ultrapure water were mixed with 5.6 g cetyltrimethylammonium bromide (CTAB) (Sigma Aldrich, Darmstadt, Germany), with vigorous stirring at ambient temperature until a clear solution was obtained. The supernatant solution containing previously obtained sodium silicate was dropwise added to the aqueous CTAB solution and stirred for 2 h. The pH of the solution was measured, being at the top limit of the basic scale (14) and it was corrected to 10.5 with concentrated sulfuric acid and the mixture was aged overnight. Finally, pH was again verified and then the gel was poured into a Teflon autoclave (PARR Instrument Company, Moline, IL, USA) and introduced in a furnace at 373 K for 48 h so that the hydrothermal reaction can be achieved. The mixture was then cooled and filtered. The resulted solid was dried at 378 K and then calcinated at 823 K for 6 h. The result of the calcination is a white solid of mesoporous MCM-41. The average yield was 75–80% of the resulting MCM-41 based on the raw material (fly ash), high enough for using a waste as raw material.

### 2.2. Obtaining MMMs

The membranes were obtained by applying a previously developed procedure [21]. Briefly, the polymer matrix (polysulfone–PSF, Sigma-Aldrich, Steinheim, Germany) and the filler (MCM-41-type mesoporous silica obtained from fly ash) were introduced in an oven under vacuum, at 353 K and 1.2 MPa, for 2 h, in order to remove the moisture. Subsequently, the MCM-41 material was immersed in chloroform (Sigma-Aldrich, Steinheim, Germany) and homogenized by ultrasonication (Elma S60H–Elma Schmidbauer GmbH, Singen, Germany), resulting in three concentrations: 15, 25, and 35 wt.% mesoporous silica in chloroform.

After the addition of polymer pellets, the homogeneity was assured through magnetic stirring alternated with sonication cycles. In order to obtain thin membranes and to decrease the gas flow resistance, the polymer-mesoporous silica solutions were casted in Petri dishes and left overnight for slow chloroform evaporation.

The membranes thickness was measured in 10 points by using a micrometer (Schut Geometrische Meettechniek, Groningen, The Netherlands).

After the synthesis, the membranes were degassed at 353 K and 1.2 MPa for 24 h and kept in a desiccator (Isolab Laborgerate GmbH, Eschau, Germany).

### 2.3. Characterization Techniques

The composition of the fly ash and the mesoporous silica was determined by X-ray fluorescence (XRF) spectrometry analysis, made under vacuum on a ARL PERFORM’X X-ray fluorescence (XRF) sequential spectrometer (Thermo Fisher Scientific, Waltham, MA, USA) equipped with a Rh tube as the X-ray source and 30 μm Be window.

The morphology of the silica material and the membranes was determined using a Variable Pressure Field Emission Scanning Electron Microscope-FESEM VP (Carl Zeiss, Oberkochen, Germany) with a resolution of 0.8 nm at 30 kV or 2.5 nm at 30 kV in VP mode. An available free software ImageJ (https://imagej.nih.gov/ij/) was used to calculate the particle size from SEM images. The membranes were evaluated in sections. A special treatment of the membranes was mandatory prior to SEM analysis: the membranes were immersed in liquid nitrogen and then they were broken, because a normal cut, by using a blade, would result in membrane crushing and unnoticeable arrangements of particles in the matrix could not be detected. Additionally, to assure the conductivity and the protection of the membranes, the samples were prior covered with a gold layer of approximately 20 nm thickness [21].

Brunauer–Emmett–Teller (BET) specific surface and pore diameter were determined from the N_2_ adsorption-desorption isotherms, obtained at 77 K by using an Autosorb-iQ-C instrument (Quantachrome Instruments, Boynton Beach, FL, USA). Prior to sorption analysis, the mesoporous silica was preheated to 453 K for degassing and cooled to room temperature under vacuum.

X-ray diffraction (XRD) was used in order to characterize the mesoporous MCM-41, by the means of Rigaku 600MiniFlex (Rigaku Corporation, Tokyo, Japan) with fixed Cu Kα source (λ = 0.1514 nm) and rotating silicon strip detector. 2θ scans were performed between 5° and 90°, with a speed of 1°/min and resolution of 0.015°/step.

Specific groups of the mesoporous MCM-41 were identified using Fourier transform infrared spectroscopy (FTIR) (Cary 630, ATR-FTIR (Agilent Technologies, Inc., Santa Clara, CA, USA).

Thermogravimetric determinations were performed using a SDT Q600 V20.5 Build 15 instrument (TA Instruments, New Castle, DE, USA). The weight changes were evaluated under N_2_ atmosphere (flow rate: 100 mL/min, 99.999%vol purity), at a heating rate of 10 K/min in the 303–1073 K range.

### 2.4. Gas Permeability Tests

**Figure 1:**
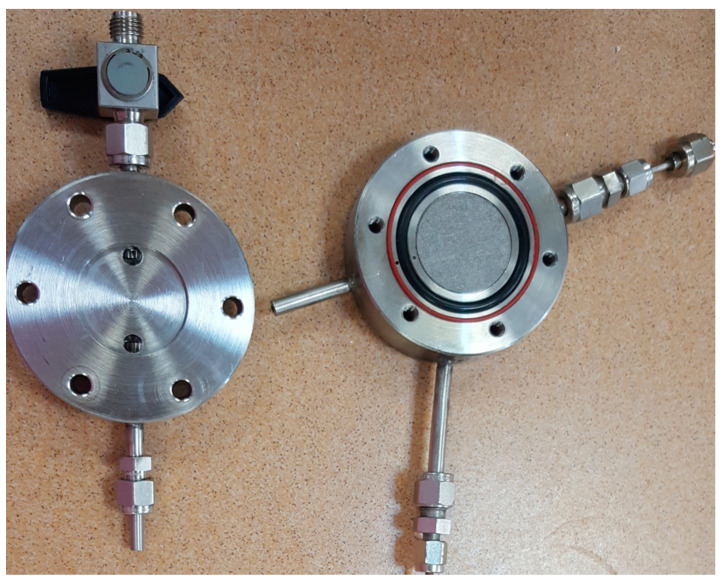
Membrane cell set-up.

The experimental set up used for membranes testing, in terms of establishing their permeabilities, was previously designed [21]. The membranes were tested with pure gases (CO_2_ and CH_4_) and with CO_2_/CH_4_ mixture (30/70), in order to compare the ideal and real selectivity, to see if they are suitable for industrial applications. The experimental set-up was modified in order to obtain an improved certainty (Figure 1).

Briefly, the main component of the installation consisted in two stainless steel plates with two O-rings between them for membrane sealed. After introducing the membrane, the stainless-steel plates were tightened with screws. The O-ring with the small diameter provided the membrane sealing and the big one allowed monitoring the potential appearance of leaks on the first O-ring during the operation. The first check of the membrane sealing was performed through vacuum pump, providing also the evacuation of the residual gases from gas channel and membrane cell. After the cleaning step, the gas mixtures feed was established at 1 bar, up to membrane chamber, and the permeation gases were on-line monitored and analyzed by using a Varian CP-3800 gas chromatograph (Varian, Walnut Creek, CA, USA) connected to the downside of the chamber through a stainless-steel channel. The permeation tests were considered finished after no changes of selectivity were observed in the permeated gas.

Three permeability tests were achieved for the same membrane in order to confirm the repeatability.

## 3. Results and Discussion

### 3.1. Mesoporous MCM-41 Characterization

The XRF elemental analysis of the samples is compiled in Table 1.

As it can be seen, the Si content of the fly ash is quite high (23.43%). The Si content of the obtained mesoporous silica is 45.54%, similar with the content of Si from MCM-41 obtained from classical synthesis.

**Figure 2:**
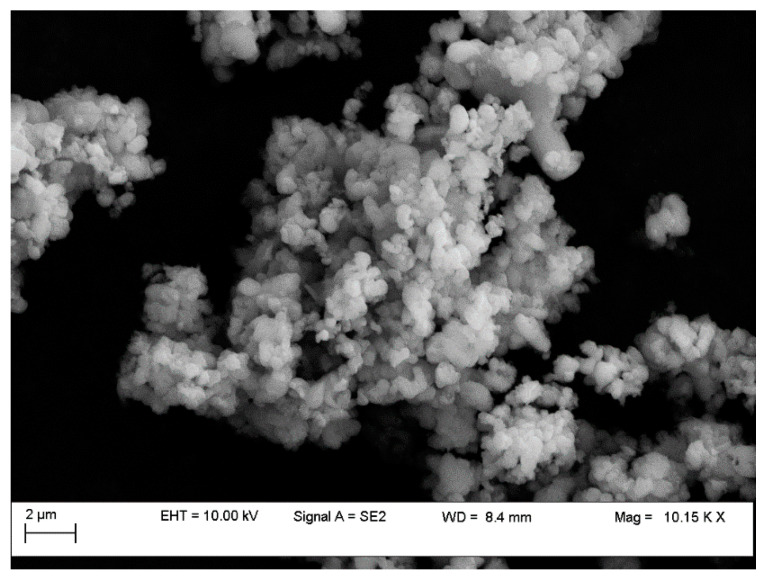
SEM image of the MCM-41 obtained from coal ash.

The resulted mesoporous material was investigated by emission field scanning electron microscopy operated at a voltage of 10 kV (Figure 2).

**Figure 3:**
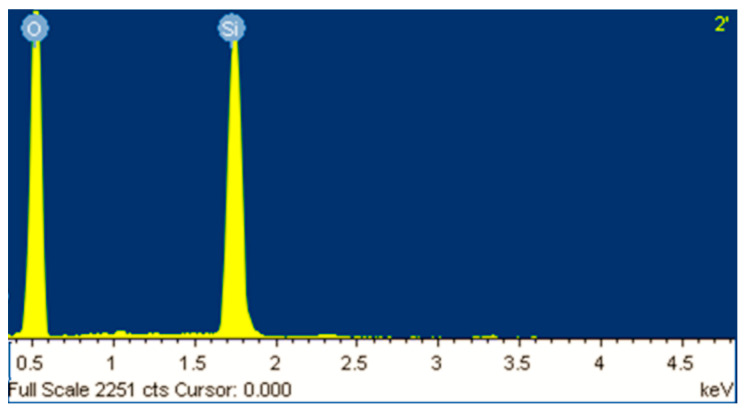
EDX analysis of the MCM-41 obtained from coal ash.

It can be observed that the obtained MCM-41 displayed a morphology approaching spherical and partially elongated shapes (with average particle size of 250 nm), similar with the morphology of typical MCM-41 synthesized from commercial silicate or wastes [12,21,22]. EDX analysis revealed strong intensity for Si and O as shown in Figure 3, confirming silica as the predominant element in the sample. The results revealed a content of about 40% by weight Si in the resulted mesoporous MCM-41 silica.

**Figure 4:**
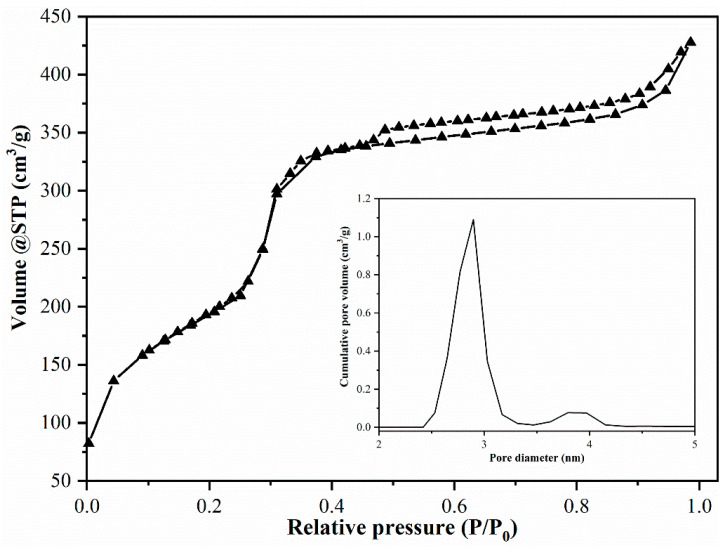
MCM-41 adsorption isotherm and pores distribution.

N_2_ adsorption–desorption isotherms (Figure 4), obtained at 77 K, correspond to a typical type IV isotherm, according to IUPAC definition.

The BET surface area for MCM-41 was 1060 m^2^/g, indicating a mesoporous material, similar to MCM-41 obtained when using commercial silicate as silica source [20]. The BJH method revealed an average pore diameter of 2.89 nm (Figure 4).

**Figure 5:**
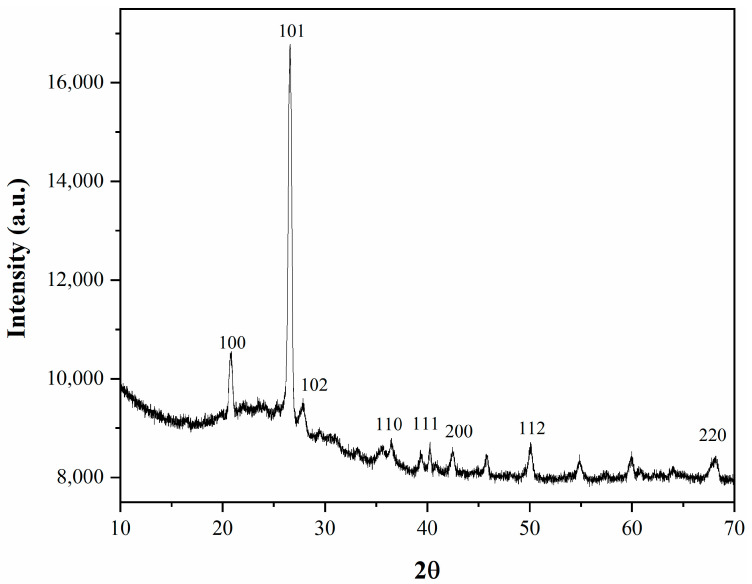
XRD for the MCM-41 obtained from coal ash.

Figure 5 shows the X-ray diffraction for the silica resulting from the coal ash.

The diffraction peaks of 2θ = 21°, 27°, 28°, 31°, 36.5°, 40°, 42.5°, 50°, and 68° indicated the presence of SiO_2_ (01-075-3168), crystobalite alpha (01-080-3753), and synthetic crystobalite (00-011-0695) with hexagonal P41212, P3221, and hexagonal P41212 crystal structures symmetries. In the crystobalite alpha form, the lattice constants (a, b) were 4.743 Å, and c constant was 6.289 Å. For the synthetic form of crystobalite, the lattice constants had slightly increased values, a, b being 4.834 Å and c had also an increased value of 6.835 Å. For the silica phase a, b was 3.260 Å and c was 4.340 Å. The phase of silica (SiO_2_) is a highly ordered superstructure having the lattice constants a, b equal (a = b = 3.260 Å) and high lattice constant value c of 10.610 Å. All the identified phases were in accordance with the previous XRD results, with the difference that the constitutive phases were detected in various proportions, XRD peaks being more or less pronounced [23].

**Figure 6:**
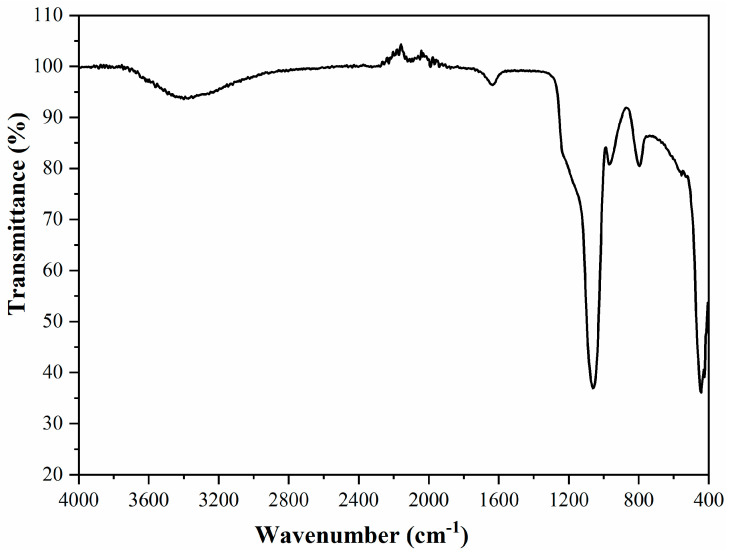
FTIR spectra of the mesoporous MCM-41.

Figure 6 presents the FTIR spectra of the obtained MCM-41.

The CTAB was completely eliminated during calcination, the proof being the absence of the peaks corresponding to d-C–H vibrations [24,25]. The bandwidth from 3400 cm^−1^ appeared due to the surface silanols and the adsorbed water molecules. The peak from 1636 cm^−1^ was assigned to the deformation mode of the hydroxyl group existent on the silica surface. The band at 1060 cm^−1^ was attributed to the asymmetric stretching of the Si–O–Si. The band at 790 cm^−1^ was due to the Si–O–Si bending and the band at 450 cm^−1^ was due to the Si–O–Si rocking [26,27]. At 965 cm^−1^ a band appeared due to the stretching of Si–OH and Si–O– groups from the surface, the respective band had the weak broad shoulder at about 540 cm^−1^ [25].

**Figure 7:**
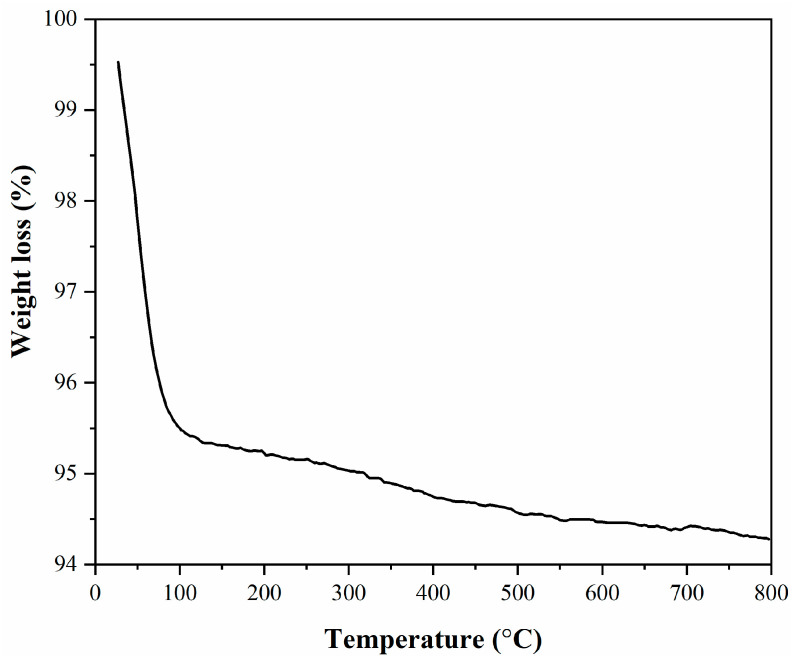
TG analysis of the mesoporous MCM-41.

TG analysis of the mesoporous MCM-41 (Figure 7) revealed two intervals of weight loss, the first one being caused by the loss of the water from the silica surface, the second one being assigned to the mesoporous structure disruption [21,28].

The total mass loss for the MCM-41 was about 5.25%.

### 3.2. Membrane Characterization

**Figure 8:**
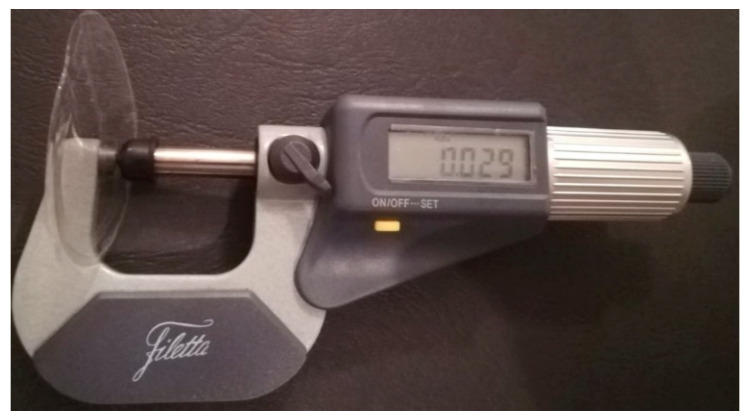
MMMs thickness.

The micrometer measurements revealed that the obtained membranes had a mean thickness around 30 µm (Figure 8).

**Figure 9:**
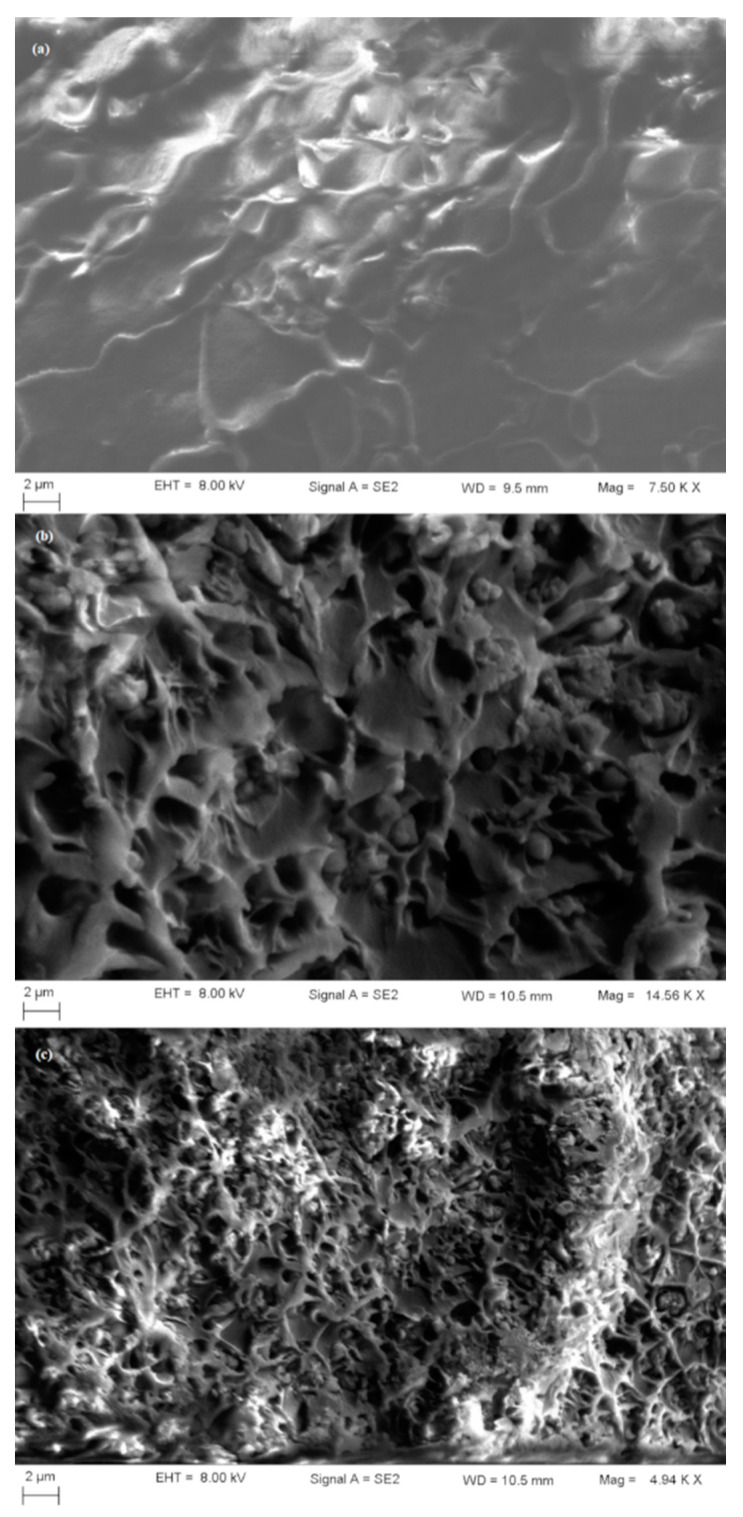
SEM image of the membranes ((**a**)—15% MCM-41/PSF; (**b**)—25% MCM-41/PSF; (**c**)—35% MCM-41/PSF).

Taking into account the resulting SEM cross-section micrograph images (Figure 9a–c) of the membranes, it seems that the mesoporous particles were well dispersed in the polymer matrix and no cracks were identified. Additionally, there were observed silica nanoparticles agglomerations, forming probably some unselective channels. These agglomerations were more pronounced in the case of the membranes obtained with higher quantities of particles, due to the presence of hydrogen bond. In the neat membrane (polymer only) and 15% MCM-41/PSF (Figure 9a) no gaps formation was observed, but they appeared in the case of 25% MCM-41/PSF (Figure 9b) and 35% MCM-41/PSF (Figure 9c), even if the penetration of polymer chain was achieved.

**Figure 10:**
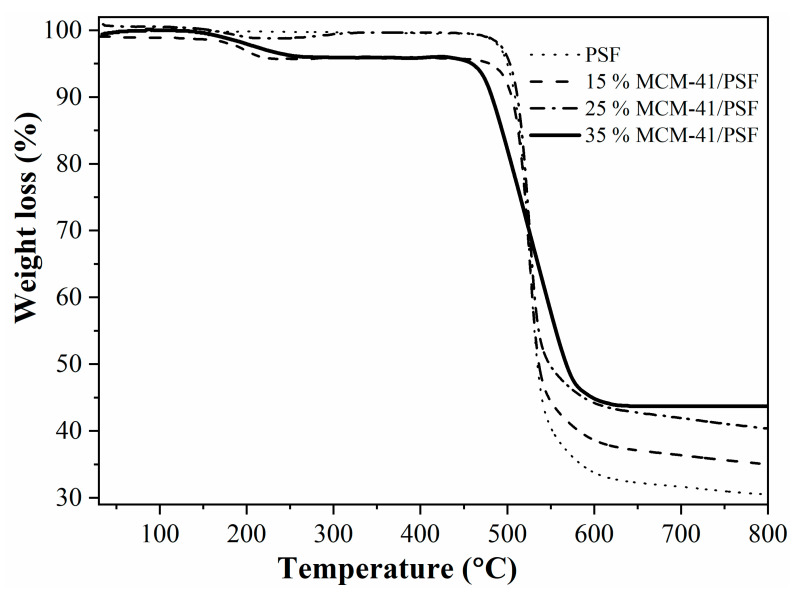
TG analysis of the membranes.

Figure 10 presents the thermogravimetric curves for the synthesized membranes, indicating a small weight loss up to 503 K (230 °C), confirming the loss of residual water or organic solvent [29,30].

The polymer chain disruption occurred after 723 K (450 °C), proving that the membrane is thermally stable up to this temperature. The residues weights obtained up to 1073 K (800 °C) were directly proportional with the silica filler, confirming that the membranes were homogenous.

The tendency of the first weight loss was similar especially for 15% and 35% filler, but at the final of the analysis the more thermal stable membrane was the 35% MCM-41/PSF followed by 25, 15, and PSF membranes. The thermal stability increased with the filler loading due to formation of inorganic–organic nanocomposites. Water content was correlated with the membrane hydrophilic property [30]. It was obvious that the water content increased by increasing the weight percent of the MCM-41 filler. Disjunction of the polymer chain due to the filler created spaces in the PSF matrix, leading to water content increase.

**Figure 11:**
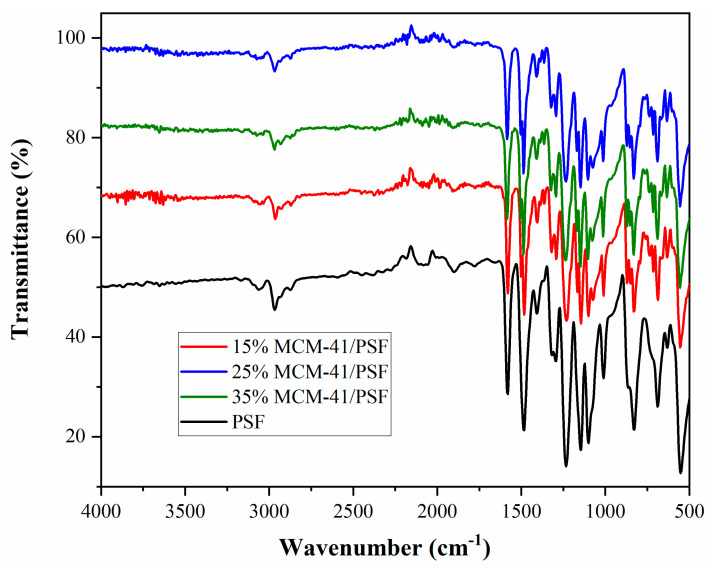
FTIR of the membranes.

Figure 11 presents FTIR spectra for the neat membrane and for the MMMs with 15–35 wt.% silica.

In the MMMs spectra, the vibration band characteristic to Si–O–Si, at 1100 cm^−1^ is covered by the intensity of the polysulfone matrix [31]. Additionally, the typical bands for polysulfone matrix were observed: at 831 cm^−1^ attributed to C–H bond; at 1010 and 1100 cm^−1^ assigned to C–C bond; at 1144 and 1315 cm^−1^ attributed to Ar–SO_2_–Ar (Ar–aromatic); at 1233 cm^−1^ assigned to Ar–O–Ar stretching [32]. The MCM-41 insertion influenced the intensity of the bands presented in the matrix.

### 3.3. Membrane Testing/Gas Permeation

For gas permeation test, pure gases and gas mixture (CO_2_/CH_4_) were used in order to determine the ideal and real selectivity. All membranes were tested under the same conditions, at 1 bar feed pressure and 297 K. The neat membrane was obtained using only polysulfone polymer dispersed in the same solvent, without adding silica filler.

**Figure 12:**
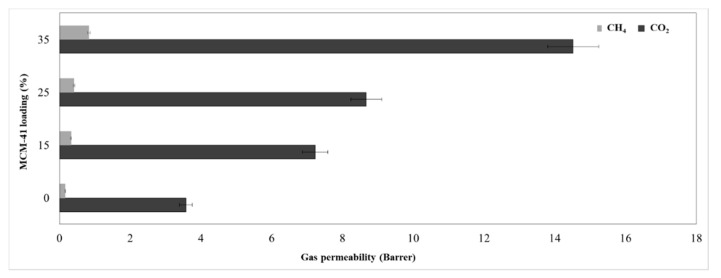
Membranes permeability.

**Figure 13:**
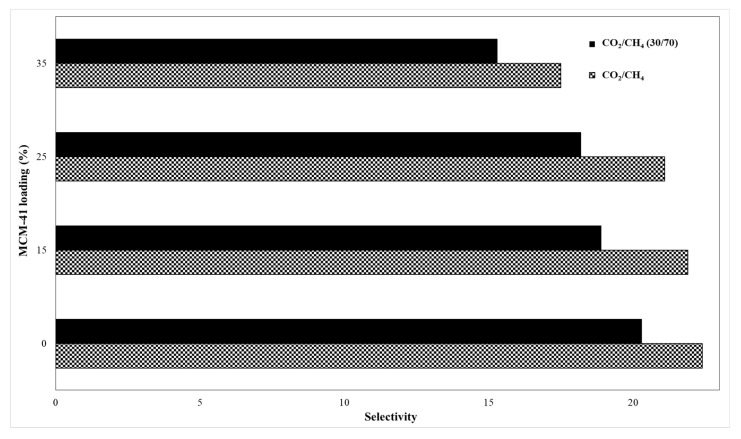
Membranes ideal and real selectivity.

Figure 12 shows the permeability for pure gases (CO_2_ and CH_4_), whereas Figure 13 presents their ideal versus real selectivity. The error for the CO_2_ and CH_4_ permeability was ±5%.

In addition, the obtained data were introduced in Table 2 to better evaluate the differences between ideal and real selectivity.

The kinetic diameter difference between the two gas molecules, namely CH_4_ (3.8 Å) and CO_2_ (3.3 Å) is considered an important vector for the mixed matrix membrane separation by sieving mechanism, while the gas separation mechanism for polymeric membranes is based on solution-diffusion of gas molecules [2]. Therefore, the increase of permeability is justified by the increase of silica loading, because the gas molecules will not meet high resistance through the formed mesoporous channels. Thus, the CO_2_ permeability for the 35% MCM-41/PSF membrane was over four times higher comparing with the neat membrane, increasing from 3.58 to 14.52 Barrer. A small difference for CO_2_ permeability was observed in the case of 15% and 25% MCM-41/PSF, which were about two times higher than the PSF neat membrane. Regarding the CH_4_ permeability values obtained for all silica-filled membranes, these were significantly lower, contributing to the idea that the membranes act as selective barriers. As it was expected, the most selective membrane was the neat membrane, but no significant changes were observed for 15% and 25% MCM-41/PSF. A noticeable loss of selectivity was remarked for 35% MCM-41/PSF, from 22.4 (for the neat membrane) to 17.5, but this can be sustained taking into account the high quantity of filler in the membrane, contributing to particles agglomeration and, consequently, to small disruption of the polymer chain and unselective voids appearance.

The performance of the obtained membranes was also evaluated by using a CO_2_/CH_4_ mixture (30/70) in order to assess the effect of the second gas on the selectivity, by its interaction with the membrane materials or both gases penetrant competition.

Regarding the calculated selectivity, it can be highlighted a small difference between the values, the real selectivity slightly decreasing, indicating that the synthesized membranes exhibit the separation performances even with gas mixture.

**Figure 14:**
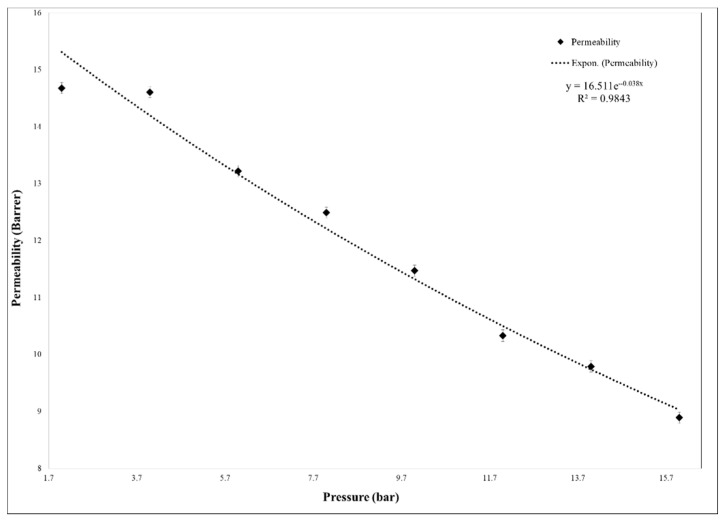
Membranes plasticization effect.

The membrane plasticization caused by CO_2_ was also investigated through several permeability tests with pure CO_2_ at different feed pressures, from 2 to 16 bar. The 35% MCM-41/PSF membrane was subjected to permeability tests and the permeability evolution is represented in Figure 14.

**Figure 15:**
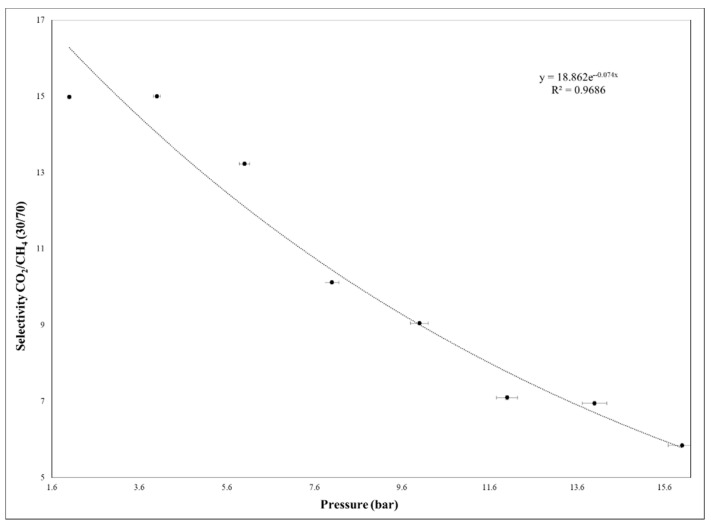
The 35% MCM-41/PSF selectivity at different feed pressure by using gas mixture.

Furthermore, in order to demonstrate their industrial processes applicability and for a better comparison, the plasticization effect was also monitored by using gas mixture (30/70%, CO_2_/CH_4_) (Figure 15).

The CO_2_ permeability remained almost constant up to 4 bar and decreased up to 16 bar, suggesting that the plasticization is missing, because this phenomenon typically causes the increase of permeability with the pressure increasing [33,34,35]. Additionally, in the case of gas mixture test, the membrane showed high resistance and the same tendency was observed as in the case of single gas testing. As it was demonstrated in other studies, the plasticization effect of neat PSF membrane appeared after high feed pressure of approximately 30 bar [34]. Therefore, the synthesized 35% MCM-41/PSF membrane presented high resistance to plasticization, being available for processes which work at high pressures. Once the feed pressure increases, it is obvious that the efficiency of membranes would decrease, being also suitable for processes which release carbon dioxide in atmosphere, such as fuel combustion.

**Figure 16:**
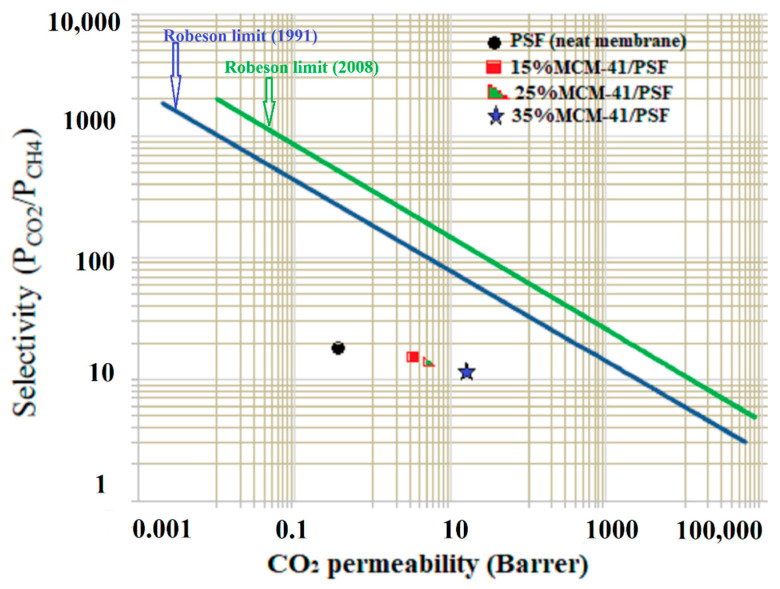
Robeson’s plot of CO_2_/CH_4._

The obtained data for CO_2_/CH_4_ were evaluated against the Robeson limit [36]. The general trade-off between permeability and selectivity was first quantified by Robeson in 1991, when he identified upper bounds in plots of log (P*_x_*/P*_y_*) versus log P*_x_* for gas pairs based on the gas permeability of the best performing polymers at that moment. In 2008, Robeson updated all of the upper bounds using initial data for two spirobisindane-based polymers, having rigid and contorted macromolecular structures that provided very good high permeability with moderate selectivity. Figure 16 compares the effectiveness of the membranes over the pure PSF membrane (neat membrane) for permeability of gases.

As it can be observed, the values are below the Robeson upper bound, but this fact does not mean that the membranes could not be applicable for natural gas separation, these new membranes having a potential significant contribution to the understanding of gas transport in mesoporous fillers and sulfonated polymers based MMMs. The data points for the membranes were in the region of conventional polymeric membranes, their separation performance being lower than that of thermally rearranged (TR) polymeric membranes or zeolite membranes [36]. Furthermore, most of the polymeric membranes performing in the region close to upper-bound have other drawbacks (such as CO_2_ plasticization even when low pressures were applied) [29].

Future optimization of obtaining parameters for these membranes could potentially drive to more CO_2_ selective ultimately extremely competitive membranes. Additionally, as it was previous demonstrated, by functionalizing the fillers with suitable amino groups, the separation performances of the membrane could be improved, due to the enhancement of the interfacial asset between the filler and the polymer matrix [21,37].

The performances of the obtained MMMs are comparable with other membranes developed from various fillers and matrix, as it can be seen in Table 3.

It can be observed that, in the case of the MMMs obtained from metal doped MOF-5 and polyimide (PI), the permeability and the selectivity was significantly lower, even at lower filler loadings and despite the higher active surface of MOFs versus MCM-41 [38]. When comparing with similar membranes based on MCM-41 (synthesized from commercial materials) as filler and PSF as matrix, tested in different conditions [39,40], it can be remarked that the results were in the same range with the membranes obtained in this study, below the Robeson upper limit. Although the CO_2_ permeability was significantly higher for 4A Zeolite/Pebax-1657 membranes for all loadings at higher testing pressure (4.9 Bar), the selectivity was lower for 20% and 30% loading [41], comparing with the values obtained in the present study (Table 1).

## 4. Conclusions

MMMs have gained over the last decade intense interest due to their potential to surpass the performance trade-off manifested by the traditional polymeric membranes. MMMs were easily synthesized using mesoporous silica as filler. In order to obtain the silica mesoporous material, coal fly ash was used as raw material. The characterization techniques proved that the materials have similar morphology with a typical MCM-41-silica material. The MCM-41 from fly ash presented large surface area and narrow ordered pores, which constitutes advantages in gas separation performances when it is introduced in a polysulfone matrix.

The use of ordered mesoporous silica MCM-41 to manufacture MMMs not only enhanced the filler dispersion and interaction with the polysulfone (highlighted by SEM, TG and FTIR) but also induced a significant augmentation of the separation performance. The tests illustrated that the mixed membranes manifest better gas separation qualities than a neat polysulfone membrane.

The permeability experiments and determination of real and ideal selectivity showed encouraging results on the application of these membranes in industrial processes, such as natural gas purification. Slight differences were observed between the real and ideal selectivity, the membranes efficiency prevailing even with CO_2_/CH_4_ mixture (30/70).

## Figures and Tables

**Table 1:** XRF data of the fly ash and MCM-41 obtained from coal ash.

Element	Fly Ash (%wt.) *	Est. Error	MCM-41 (%wt.) **	Est. Error
Si	23.4300	0.1200	45.5400	0.0400
Al	14.6500	0.1200	0.0670	0.0054
Fe	6.7200	0.1000	0.0178	0.0009
K	3.1100	0.0800	0.0176	0.0010
Ca	2.6200	0.0700	0.0077	0.0006
Mg	1.0800	0.0400	-	-
Ti	0.7390	0.0300	-	-
S	0.3500	0.0170	-	-
Na	0.4210	0.0310	0.6300	0.0310
P	0.0953	0.0048	-	
Ba	0.0883	0.0044	-	
Mn	0.0551	0.0028	-	
V	0.0276	0.0014	-	
Sr	0.0409	0.0020	-	
Cr	0.0268	0.0013	-	
Zr	0.0211	0.0011	-	
Ni	0.0195	0.0010	-	
Zn	0.0194	0.0010	-	
Rb	0.0178	0.0009	-	
Cu	0.0131	0.0007	-	
Ce	0.0116	0.0024	-	

* Total O (%wt.) = 53.0700; ** Total O (%wt.) = 46.4000.

**Table 2:** Permeability and selectivity results of the membranes obtained with pure and gas mixture.

Silica Loading wt.%	Permeability, Barrer		Ideal Selectivity (P_CO2_/P_CH4_)	Real Selectivity (CO_2_/CH_4_–30/70%)
	CO_2_	CH_4_		
0	3.58 ± 0.18	0.16 ± 0.01	22.4	20.3
15	7.23 ± 0.37	0.33 ± 0.02	21.9	18.9
25	8.67 ± 0.43	0.41 ± 0.02	21.1	18.2
35	14.52 ± 0.73	0.83 ± 0.04	17.5	15.3

**Table 3:** Different MMMs performances.

Mixed Matrix Membrane	Permeability (Barrer)		Ideal Selectivity (PCO_2_/PCH_4_)	Testing Condition	Reference
	CO_2_	CH_4_			
5% MOF-5/PI	~3	~0.4	~7.5	1 Bar, 298 K	[38]
5% CuMOF-5/PI	~4	~0.5	~8.0	1 Bar, 298 K	[38]
5% CuCoMOF-5/PI	~4.2	~0.5	~8.4	1 Bar, 298 K	[38]
10% MCM-41/PSF	10.5	0.6	17.5	2 Bar, 298 K	[39]
20% MCM-41/PSF	11.4	0.6	19.0	2 Bar, 298 K	[39]
30% MCM-41/PSF	20.5	1.0	20.5	2 Bar, 298 K	[39]
10% MCM-41/PSF	6.6	0.3	22.0	4.04 Bar, 308 K	[40]
20% MCM-41/PSF	7.8	0.3	26.0	4.04 Bar, 308 K	[40]
40% MCM-41/PSF	14.8	1.0	14.8	4.04 Bar, 308 K	[40]
5% 4A Zeolite/Pebax-1657	71.4	2.2	32.5	4.9 Bar, 308 K	[41]
10% 4A Zeolite/Pebax-1657	97.0	3.7	26.2	4.9 Bar, 308 K	[41]
20% 4A Zeolite/Pebax-1657	113.7	6.5	17.5	4.9 Bar, 308 K	[41]
30% 4A Zeolite/Pebax-1657	155.8	19.7	7.9	4.9 Bar, 308 K	[41]

## Data Availability

The raw/processed data required to reproduce these findings cannot be shared at this time due to legal or ethical reasons.
